# Comparing the Similarity of Different Groups of Bacteria to the Human Proteome

**DOI:** 10.1371/journal.pone.0034007

**Published:** 2012-04-25

**Authors:** Brett Trost, Rolando Pajon, Teenus Jayaprakash, Anthony Kusalik

**Affiliations:** 1 Department of Computer Science, University of Saskatchewan, Saskatoon, Saskatchewan, Canada; 2 Center for Immunobiology and Vaccine Development, Children’s Hospital Oakland Research Institute, Oakland, California, United States of America; 3 Department of Veterinary Microbiology, University of Saskatchewan, Saskatoon, Saskatchewan, Canada; Saint Louis University, United States of America

## Abstract

Numerous aspects of the relationship between bacteria and human have been investigated. One aspect that has recently received attention is sequence overlap at the proteomic level. However, there has not yet been a study that comprehensively characterizes the level of sequence overlap between bacteria and human, especially as it relates to bacterial characteristics like pathogenicity, G-C content, and proteome size. In this study, we began by performing a general characterization of the range of bacteria-human similarity at the proteomic level, and identified characteristics of the most- and least-similar bacterial species. We then examined the relationship between proteomic similarity and numerous other variables. While pathogens and nonpathogens had comparable similarity to the human proteome, pathogens causing chronic infections were found to be more similar to the human proteome than those causing acute infections. Although no general correspondence between a bacterium’s proteome size and its similarity to the human proteome was noted, no bacteria with small proteomes had high similarity to the human proteome. Finally, we discovered an interesting relationship between similarity and a bacterium’s G-C content. While the relationship between bacteria and human has been studied from many angles, their proteomic similarity still needs to be examined in more detail. This paper sheds further light on this relationship, particularly with respect to immunity and pathogenicity.

## Introduction

Microorganisms and their hosts have complex relationships that are still not completely understood. Given the ramifications for health and disease, the microbe-human relationship is of particular interest. Numerous aspects of this relationship have been studied, including pathogenesis, virulence, immunity, mutualism/commensalism, and metabolism.

Recently, there has been increasing interest in the level of protein sequence overlap between microbes and human. Much of this interest has been motivated by what we will call the “similarity hypothesis” [1], [2], whose assumptions are based on the classical view of the adaptive immune response. According to this view, self-tolerance is achieved by deletion or anergy of immature self-reactive B- and T-cells–a process called negative selection. Negative selection suggests that the immune system would be unlikely to recognize foreign peptide segments that are sufficiently similar to self-peptides, as B- and T-cells specific for these peptides would be deleted or rendered anergic. This leads to the hypothesis that similarity to the host proteome may play an important role in determining whether a potential protein antigen will be recognized by the immune system–or more specifically, that a peptide segment that is found rarely or never in the host proteome is more likely to be immunogenic than a peptide segment that is found many times in the host proteome.

The similarity hypothesis predicts that epitopes will be less similar to the host proteome than will non-epitopes. This prediction has been examined in a number of experimental studies (see [3] and references therein). For instance, Rolland and colleagues found an inverse relationship between the immunogenicity of HIV-derived peptides and their similarity to the human proteome [4], and Amela and co-authors discovered that B-cell epitopes derived from pathogens have lower sequence similarity to the human proteome than would be expected by chance [5].

Louzoun et al. made another prediction stemming from the similarity hypothesis–that viruses may experience selective pressure to express proteins segments that are identical to ones in their host in order to escape the host’s immune response [6]. They found that the degree of overlap between the human proteome and the proteomes of viruses infecting humans was significantly higher than expected. More recently, it was discovered that viruses infecting humans have fewer peptides likely to be recognized by the human immune system than do viruses infecting non-human hosts [7]. The same authors also found that proteins expressed early in the viral life cycle contain fewer epitopes than proteins expressed later, perhaps giving the virus time to replicate before a viable immune response can be initiated. Combined, these observations suggest that viruses experience selective pressure to eliminate immunogenic epitopes from the proteins that they express.

There are two primary differences between this study and the ones cited in the previous paragraph. First, bacteria are examined rather than viruses. Second, instead of focusing exclusively on immunogenicity and pathogenicity, we conduct a broad examination of bacterial properties and determine if they have a relationship with bacteria-human proteome similarity. The specific aspects of bacteria-human similarity that are examined in this study are described below.

First, we report a general characterization of bacteria-human similarity among all 975 bacteria whose genome sequences were available at the time of this study. Next, three predictions stemming from the similarity hypothesis are examined. First, we predict that the proteomes of pathogenic bacteria are more similar to the human proteome than those of nonpathogenic bacteria, since pathogenic bacteria may experience evolutionary pressure to modify their proteins so that they become more similar to the host (i.e. human) in order to eliminate potentially immunogenic epitopes. Second, for analogous reasons we predict that proteins that are accessible to the immune system are more similar to the human proteome in pathogens than in nonpathogens. Third, we predict that the proteomes of bacteria causing chronic infections are more similar to the human proteome than those of bacteria causing acute infections, as the former group seems better able to resist immune responses. After evaluating these predictions stemming from the similarity hypothesis, we also examined other variables that may be related to a given bacterium’s similarity to the human proteome–specifically, proteome size, G-C content, and (for pathogens) mode of transmission.

## Methods

### Comparing the Similarity of Bacterial Proteomes to the Human Proteome

Most of the analyses in this paper compare the similarity to the human proteome of different bacterial proteomes or sets of proteomes. One analysis compares the similarity of different sets of proteins (rather than proteomes), but the procedure is the same; for brevity, this section uses “proteomes” to refer to both actual proteomes and defined sets of proteins. The different sets of proteomes and proteins used are described in subsequent sections.

The technique used to compare the similarity of bacterial proteomes to the human proteome has been described previously [8], but is reiterated here. As peptide segments of length five (5-mers) have been described as fundamental units for immunological recognition and protein-protein interactions [9], to measure bacteria-human similarity we determined the percentage of 5-mers in different bacterial proteomes that are found a certain number of times in the human proteome. To do this, each protein from each bacterial proteome was decomposed into all possible 5-mers; for example, a protein of 150 amino acids was decomposed into 146 5-mers. It was then determined how often each 5-mer was found in the human proteome. If the same 5-mer was found more than once in a given bacterial proteome, then each instance was counted. For example, if a given 5-mer (say, ACDEF) was found ten times in a particular bacterial proteome, and that 5-mer was found zero times in the human proteome, then this increased by ten the number of 5-mers in that bacterial proteome that were found zero times in the human proteome. This would have the same effect as if ten distinct 5-mers were each found one time in the bacterial proteome, and each of these ten 5-mers were found zero times in the human proteome.

Bacterial proteomes with few 5-mers that were found rarely or never (as exact matches) in the human proteome were described as “similar” to the human proteome, and vice versa. Since only exact matches of 5-mers were considered, here the word “similarity” does not refer to the similarity of individual sequences (such as two 5-mers being identical except for one amino acid substitution), but rather to overall proteomic similarity as measured by the frequency of bacterial 5-mers found rarely or never in the human proteome. For simplicity, we collectively called 5-mers that are found rarely or never in the human proteome “rare 5-mers”.

Because there is no standard definition of a rare 5-mer, we used three possible definitions: a 5-mer occurring zero times in the human proteome, a 5-mer occurring two or fewer times, and a 5-mer occurring five or fewer times. Whenever a statistical test was performed, it was done for each of these definitions. However, some analyses not involving statistical tests were done only for the zero-times definition.

In a previous paper examining the level of self-similarity of human proto-oncoproteins to the human proteome [8], we also performed the above procedure using 6-mer and 7-mer peptides, and found that the results were uniformly consistent with those obtained when using 5-mers. That is, if one set of proteins contained more rare 5-mers than another set, then it also contained more rare 6-mers and 7-mers. However, most 6-mers, and nearly all 7-mers, were found very few times in the human proteome, making the results using these lengths difficult to interpret. We also noted that most 4-mers were found many times in the human proteome–very few were “rare”. Informal experiments using 4-mers, 6-mers, and 7-mers revealed similar patterns when comparing bacterial proteomes to the human proteome. As such, we limited our analyses in this paper to 5-mers.

The similarity of each individual bacterial proteome to the human proteome was analyzed as follows. As mentioned above, we determined the number of times that each 5-mer in the bacterial proteome was found in the human proteome. From this, we calculated 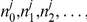 where 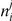 indicates the number of 5-mers in bacterial proteome *j* that occurred *i* times in the human proteome. For example, 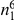 denotes the number of 5-mers in bacterial proteome #6 that occurred once in the human proteome. We also calculated 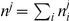
–the total number of 5-mers in bacterial proteome *j*. Finally, let 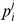 denote the percentage of 5-mers in *j* that were found *i* times in the human proteome. Then 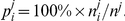 For example, suppose that there were a total of 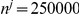 5-mers in the proteome for bacterium *j*, and that 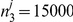 of these were found three times in the human proteome. Then the percentage of 5-mers in *j* that were found three times in the human proteome would be 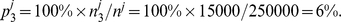


As described in subsequent sections, various “sets” were created, each consisting of several individual bacterial proteomes–for example, a set of pathogens, a set of nonpathogens, etc. The similarity of these sets of bacteria to the human proteome was visualized as described in the next section. Further, statistical tests were performed among different sets to determine if they had significantly different similarity to the human proteome; these tests are described in the “Statistical tests” section.

### Visualizing the Similarity of Sets of Bacteria to the Human Proteome

Visualizing the similarity of a given set of bacterial proteomes to the human proteome was done as follows. As described above, for each bacterium the quantities 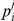 were calculated, which represent the percentage of 5-mers in bacterium *j* found *i* times in the human proteome. For all *b* bacteria in a given set, the average percentage was calculated for each *i*: 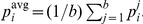 The standard deviation of these values was also calculated: 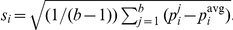 A scatterplot was then created where each point 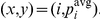 In other words, the *x*-value of each point represents the number of times a 5-mer is found in the human proteome, and the corresponding *y*-value represents the average percentage of 5-mers that are found that many times among all the bacteria in the set. Each point also has an associated error bar, which has a length in each direction equal to 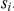 For a given scatterplot, the above was performed for two or more sets of bacterial proteomes.

### Statistical Tests

Statistical tests were used to determine whether sets of bacterial proteomes had significantly different similarity to the human proteome. As mentioned above, we used three operational definitions of a rare 5-mer: one found zero times in the human proteome, one found two or fewer times, and one found five or fewer times. For the first definition, the observations for a given set are simply the values 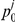 for 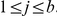 For the second and third definitions, the observations are 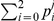 and 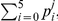 respectively, again for 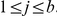


**Figure 1:**
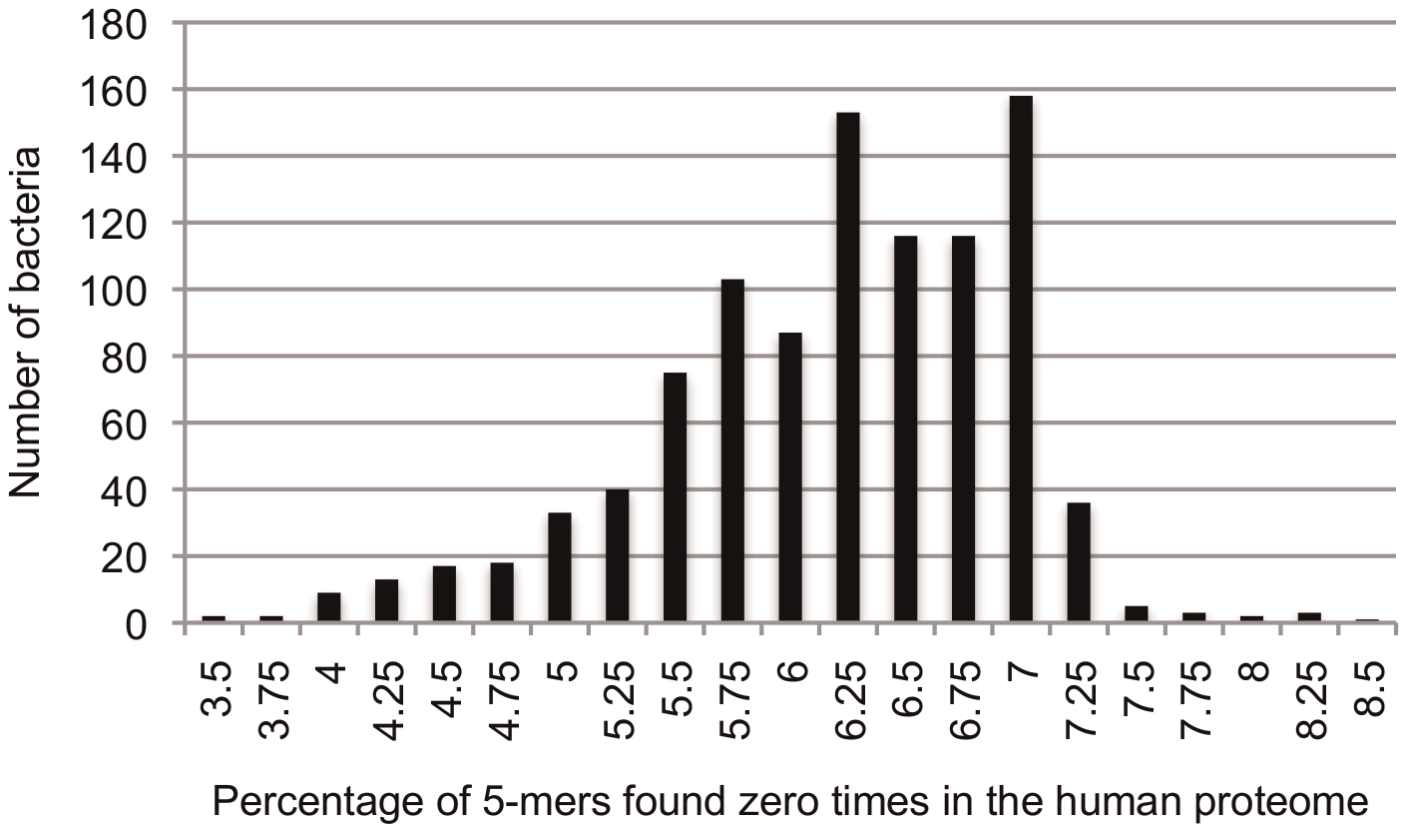
Distribution of bacterial similarity to the human proteome. All bacteria in set 1 were used in creating this histogram. Each bar represents the number of bacteria having between *x*% and 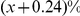 of their 5-mers not found in the human proteome, where *x* is the number below the bar.

As an initial analysis, we created a histogram showing the distribution of bacterial similarity to the human proteome among all 975 bacteria whose sequences were available when this study began (see the section “Characterizing the range of bacterial similarity to the human proteome” below). This histogram (see Figure 1) showed a distribution that deviated from normality; in particular, the tails had dissimilar lengths. As such, rather than using t-tests (for two sets) and ANOVA (for three or more sets) to determine whether groups of sets had significantly different frequencies of rare 5-mers, we used their non-parametric equivalents: the Wilcoxon rank-sum test (also called the Mann-Whitney U test) and the Kruskal-Wallis test, respectively. Two-sided, unpaired Wilcoxon rank-sum tests were performed using the R [10] function wilcox.test, while Kruskal-Wallis tests were performed using the function kruskal.test.

### Human and Bacterial Proteomes

The UniProtKB version of the human proteome was downloaded from Integr8 [11], and contained 38,009 protein sequences. All 975 bacteria that were listed on the European Bioinformatics Institute (EBI) bacteria page (www.ebi.ac.uk/genomes/bacteria.html) at the time of this study were downloaded from Integr8. The set of all bacteria will be denoted set 1; a list of these bacteria is available in Table S1. Different subsets of set 1 were also used in this study, and are described in subsequent sections.

### Characterizing the Range of Bacterial Similarity to the Human Proteome

To provide a general characterization of the range of bacterial similarity to the human proteome, we first calculated some simple descriptive statistics on all 975 bacteria in set 1. Specifically, we calculated the percentage of 5-mers not found in the human proteome for each individual bacterium in this set, and then computed the mean and standard deviation of these values, as well as a histogram showing their distribution. Finally, lists of the ten most similar and ten least similar bacteria to the human proteome were created, which were then analyzed to identify any commonalities or interesting characteristics of the bacteria contained therein.

### Comparison between Pathogenic Bacteria and Nonpathogenic Bacteria

This section describes the methodology used to compare the relative similarity to the human proteome of three sets of bacteria: pathogens (set 2, described below), nonpathogens (set 3, described below), and (as a baseline) bacteria in general (set 1, described above).

The set of bacterial pathogens (set 2) was a subset of set 1 that contained bacteria that are pathogenic to humans. Bacteria requiring level two or higher biosafety practices according to the Public Health Agency of Canada (www.phac-aspc.gc.ca/lab-bio/res/psds-ftss/index-eng.php) were candidates for inclusion in set 2. To ensure adequate phylogenetic diversity, only one species of a given genus was chosen. A list of the 20 bacteria chosen for inclusion in set 2 is given in Table 1.

**Table 1:** Pathogenic bacteria used in this study (set 2).

Species	Disease	Mode of transmission	Proteins
**Bacteria causing acute infections (set 2 a)**			
*Bacillus anthracis* (Sterne)	Anthrax	Contact with infected animals	5284
*Bartonella bacilliformis* (ATCC 35685)	Oroya fever	Sandfly bites	1255
*Burkholderia mallei* (ATCC 23344)	Glanders	Contact with infected animals	4797
*Campylobacter jejuni* (RM1221)	Campylobacteriosis	Food; contact with animals	1836
*Clostridium tetani* (E88)	Tetanus	Breaks in skin	2415
*Corynebacterium diphtheriae* (ATCC 700971)	Diphtheria	Respiratory/physical contact	2264
*Coxiella burnetii* (RSA 493)	Q-fever	Inhalation around animal reservoirs	1815
*Francisella tularensis* (FSC 198)	Tularemia	Many; not person-to-person	1528
*Legionella pneumophila* (Corby)	Legionnaires’	Inhalation; not person-to-person	3202
*Leptospira interrogans* (Fiocruz L1-130)	Leptospirosis	Animal urine-infected water/food	3654
*Listeria monocytogenes* (ATCC BAA-679)	Listeriosis	Contaminated food	2844
*Streptococcus pneumoniae* (CGSP14)	Pneumonia	Person-to-person	2193
*Vibrio cholerae* (ATCC 39315)	Cholera	Contaminated food/water	3784
*Yersinia pestis* (Angola)	Plague	Many, including person-to-person	3821
**Bacteria causing chronic infections (set 2 b)**			
*Borrelia burgdorferi* (ATCC 35210)	Lyme Disease	Tick bites	1556
*Brucella abortus* (9–941)	Brucellosis	Contaminated food/water	3077
*Chlamydia trachomatis* (ATCC VR902B)	Chlamydia	Person-to-person (sexual)	884
*Haemophilus ducreyi* (ATCC 700724)	Chancroid	Person-to-person (sexual)	1694
*Mycobacterium tuberculosis* (Oshkosh)	Tuberculosis	Person-to-person (through the air)	4201
*Treponema pallidum* (SS14)	Syphilis	Person-to-person (sexual)	1028

The species name, associated disease, mode of transmission, and proteome size are listed for each bacterium. The specific strain used is indicated in parentheses after each species name. Some bacteria cause varying symptoms or diseases; as such, a representative disease was chosen for each. These bacteria constitute set 2 as described in the text, and were divided into sets 2 a and 2 b, which represent bacteria causing acute infections and bacteria causing chronic infections, respectively. Modes of transmission were derived from the Centres for Disease Control website (http://emergency.cdc.gov).

Set 3, shown in Table 2, consisted of nonpathogenic bacteria. These nonpathogens were restricted to normal flora, since they use human as a host. To identify appropriate bacteria for set 3, we utilized the list of normal flora at the Human Microbiome Project website [12]. Bacteria from this list that lacked a sequenced genome were ignored. We then examined the annotation for each remaining bacterium at the EBI genomes page (www.ebi.ac.uk/genomes/bacteria.html), and bacteria that commonly cause opportunistic infections were discarded. While all bacteria may have the potential to cause disease under some circumstances, we attempted to select bacteria with as little association with human disease as possible. As with the pathogenic bacteria, only a single nonpathogenic species was selected from a given genus, leaving a total of 14 bacteria in set 3.

**Table 2:** Nonpathogenic bacteria used in this study (set 3).

Species	Proteins	Species	Proteins
*Bacillus halodurans* (ATCC BAA-125)	4006	*Lactobacillus acidophilus* (NCFM)	1859
*Bifidobacterium longum* (NCC 2705)	1724	*Leuconostoc mesenteroides* (ATCC 8293)	2002
*Corynebacterium efficiens* (DSM 44549)	2946	*Listeria innocua* (CLIP 11262)	3018
*Desulfitobacterium hafniense* (Y51)	5014	*Micrococcus luteus* (ATCC 4698)	2207
*Eubacterium rectale* (ATCC 33656)	3545	*Parabacteroides distasonis* (ATCC 8503)	3831
*Janthinobacterium* sp. (Marseille)	3695	*Pseudomonas fluorescens* (PfO-1)	5714
*Kocuria rhizophila* (ATCC 9341)	2352	*Rhodobacter sphaeroides* (ATCC 17023)	4124

The species name and proteome size are listed for each bacterium, with the specific strain shown in parentheses. These bacteria comprise set 3 as described in the text.

To test differences in similarity between pathogens and nonpathogens among proteins with different subcellular localizations, the predicted subcellular localization for each protein from each set 2 and set 3 bacterium was downloaded from PSORTdb 2.0 [13]. Localization data were downloaded for all possible proteins, including those encoded on plasmids. The data tables downloaded included the results of several different methods of predicting localization (see [14]); however, we used only the consensus (“final localization”) column to classify each protein. Four different groups of bacteria were used for this analysis: Gram-positive pathogens, Gram-negative pathogens, Gram-positive nonpathogens, and Gram-negative nonpathogens. Gram-positive and Gram-negative bacteria were analyzed separately because they have different possible subcellular localizations. Possible subcellular localizations were “cell wall”, “cytoplasmic”, “cytoplasmic membrane”, and “extracellular” for Gram-positive bacteria and “cytoplasmic”, “cytoplasmic membrane”, “extracellular”, “outer membrane”, and “periplasmic” for Gram-negative bacteria. Proteins classified as “unknown” were discarded. For a given set–say, extracellular proteins in Gram-positive pathogens–similarity to the human proteome was determined in the same way as described in the “Comparing the similarity of bacterial proteomes to the human proteome” section, except that the pathogens were limited to the Gram-positive ones, and the proteins composing their “proteomes” were limited to those classified as (say) “extracellular” by PSORTdb. After composing these sets, each set containing pathogens (e.g. extracellular proteins in Gram-positive pathogens) was compared to its corresponding set containing nonpathogens (e.g. extracellular proteins in Gram-positive nonpathogens) to determine whether they had different similarities to the human proteome. This was done for all subcellular localizations in both the Gram-positive and Gram-negative bacteria.

### Comparison between Acute Pathogens and Chronic Pathogens

Each bacterium from set 2 (pathogens) was classified as either causing acute infections or causing chronic infections; we called these sets 2 a and 2 b, respectively. It is important to realize that the boundary between “acute” and “chronic” is not always sharp–some bacteria cause infections that are between these two classifications, and a given bacterium might cause a chronic infection in some individuals and an acute infection in other individuals. Bearing these complexities in mind, we attempted to classify each bacterium according to its most common pattern of infection. Although a slight abuse of language, for brevity we will refer to the bacteria in these subsets as “acute pathogens” or “chronic pathogens”.

In addition, we determined whether differences in similarity to the human proteome could be attributed to differences in amino acid composition by comparing the similarity to the human proteome of real bacterial proteomes with that of randomly-generated ones. To do this, we generated set 4, which consisted of 20 randomly-generated proteomes–one corresponding to each proteome in set 2. For each protein in a given set 2 proteome, a random protein of the same length was added to the corresponding set 4 proteome. Random proteins for a given set 4 proteome were constructed by choosing amino acids with probabilities equal to their frequencies in the entire corresponding set 2 proteome. For example, if glycine occurred 7% of the time in a certain set 2 proteome, then there was a 7% chance that any particular amino acid in the corresponding set 4 proteome would be glycine. After creating set 4, we verified that each set 4 proteome and its corresponding set 2 proteome had nearly identical frequencies of each amino acid. Set 4 was then divided into sets 4a and 4b, which contained random proteomes corresponding to the acute and chronic pathogens (sets 2 a and 2 b), respectively.

Although set 4 contained thousands of randomly-generated proteins, we wished to ensure that it had an unbiased composition. To achieve this, ten versions of set 4 were generated, all of which were found to give very similar results. As such, the results described later represent those of an arbitrarily-chosen version of set 4.

Finally, in order to test a second method of generating random proteomes, we generated set 5. Each proteome in this set contained the same proteins as the corresponding protein in set 2, except the EMBOSS [15] program *shuffleseq* was used to randomly shuffle the amino acids in each protein. As with set 4, set 5 was divided into sets 5a and 5b; further, ten versions of set 5 were generated, with a representative set described in the Results section.

### Examining Other Variables Possibly Related to Similarity to the Human Proteome

This section describes the methodology used to relate similarity to the human proteome to three other bacterial properties: proteome size, G-C content, and (for pathogens) mode of transmission.

#### Proteome size

To determine whether any general relationship exists between a bacterium’s proteome size and its similarity to the human proteome, a scatterplot was created wherein the *x*-value of a given point indicates the number of proteins in a particular bacterium’s proteome, and the corresponding *y*-value represents the percentage of 5-mers in that bacterium’s proteome that were found zero times in the human proteome. This was done for all 975 bacteria in set 1.

#### G-C content

To determine the overall G-C content of a given bacterium’s genes (not its entire genome), the gene set for each bacterium was downloaded in FASTA format from Integr8 [11]. The sequences of each bacterium’s genes were concatenated together (by removing the FASTA headers), and the EMBOSS [15] program *geecee* was used to determine its overall G-C content. A scatterplot similar to that described above was then created, except with the *x*-value representing G-C content rather than proteome size.

In interpreting the above scatterplot, it was also necessary to know the overall G-C content of human genes. The human gene set was downloaded in FASTA format from the Consensus Coding Sequences (CCDS) project website (http://www.ncbi.nlm.nih.gov/projects/CCDS/CcdsBrowse.cgi) [16], and its G-C content was determined in the same manner as for the bacteria.

#### Mode of transmission

Finally, we examined whether the mode of transmission of a pathogen is related to its similarity to the human proteome. The mode of transmission of each pathogen was gathered from the Centres for Disease Control website (http://emergency.cdc.gov; see Table 1), followed by a qualitative assessment of whether any patterns exist with respect to a bacterium’s mode of transmission and its similarity to the human proteome.

## Results

### Characterizing the Range of Bacterial Similarity to the Human Proteome

In order to broadly characterize the similarity of bacteria to the human proteome, this section reports some simple statistical measures for all the bacteria in set 1. Results are reported only for the “found zero times in the human proteome” definition of a rare 5-mer; however, the results were consistent when the other two definitions were used (found two or fewer times, and found five or fewer times).

Figure 1 depicts the distribution of bacterial similarity to the human proteome, and shows that there was a considerable amount of variation in the similarity of individual bacteria. The distribution was quite skewed, with a fairly long tail on the left-hand side of the histogram (bacteria with few rare 5-mers) and a short tail on the opposite side. The mean percentage of 5-mers found zero times in the human proteome among all bacteria in set 1 was 6.3%, with a standard deviation of 0.76%. These values ranged from 3.71% for *Thermus thermophilus* strain ATCC 27634 to 8.63% for *Blochmannia floridanus*. Table 3 lists the bacteria with the smallest and largest percentage of 5-mers found zero times in the human proteome, as well as the G-C content and proteome size of each.

**Table 3:** The most similar and dissimilar bacteria to the human proteome.

Bacterium	Percent	G-C content	Proteins
**Ten most similar bacteria to the human proteome**			
*Thermus thermophilus* (ATCC 27634)	3.71	0.70	2227
*Thermus thermophilus* (ATCC BAA-163)	3.72	0.70	2201
*Kineococcus radiotolerans* (ATCC BAA-149)	3.79	0.74	4674
*Geodermatophilus obscurus* (ATCC 25078)	3.98	0.74	4795
*Anaeromyxobacter dehalogenans* (2CP-C)	4.03	0.75	4345
*Actinosynnema mirum* (ATCC 29888)	4.05	0.74	6912
*Anaeromyxobacter sp.* (K)	4.05	0.75	4444
*Anaeromyxobacter dehalogenans* (ATCC BAA-258)	4.06	0.75	4461
*Acidimicrobium ferrooxidans* (DSM 10331)	4.09	0.68	1935
*Sanguibacter keddieii* (ATCC 51767)	4.09	0.72	3710
**Ten least similar bacteria to the human proteome**			
*Blochmannia floridanus*	8.63	0.29	583
*Bacteroides vulgatus* (ATCC 8482)	8.49	0.43	3982
*Blochmannia pennsylvanicus* (BPEN)	8.46	0.32	610
*Bacteroides thetaiotaomicron* (ATCC 29148)	8.39	0.44	4773
*Bacteroides fragilis* (YCH46)	8.11	0.44	4597
*Bacteroides fragilis* (ATCC 25285)	8.09	0.44	4234
*Parabacteroides distasonis* (ATCC 8503)	7.97	0.46	3831
*Eubacterium rectale* (ATCC 33656)	7.93	0.42	3545
*Eubacterium eligens* (ATCC 27750)	7.86	0.38	2761

The ten bacteria with the highest and lowest percentage of 5-mers found zero times in the human proteome are listed, along with each bacterium’s G-C content and proteome size.

### Comparison between Pathogenic Bacteria and Nonpathogenic Bacteria

**Figure 2:**
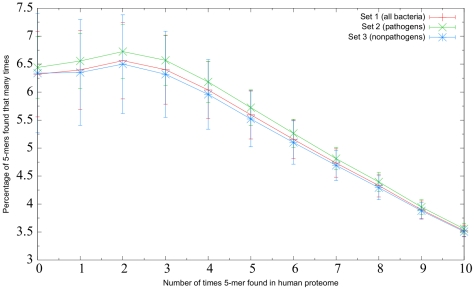
Pathogenic versus nonpathogenic bacteria. Relative similarity to the human proteome of the bacteria in set 1 (all bacteria sequenced to date), set 2 (20 pathogenic bacteria), and set 3 (14 nonpathogenic bacteria). Each point 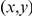 indicates that, on average, *y*% of the 5-mers in the proteomes in that set were found *x* times in the human proteome. Because only rare 5-mers are of interest in this study, bacterial 5-mers that were found more than ten times in the human proteome are not represented. The length in one direction of the error bar associated with each point represents the standard deviation of the measurements that were averaged to calculate that point.

In evaluating the similarity of different groups of bacteria to the human proteome, we first compared the relative similarity to the human proteome of 20 pathogenic bacteria (set 2) and 14 nonpathogenic bacteria (set 3). To determine a baseline level of bacterial similarity, set 1 (all bacteria sequenced to date) was also included. A plot illustrating the similarity to the human proteome of sets 1, 2, and 3 is given in Figure 2. As an example of reading Figure 2, note that except for five-mers occurring zero times in the human proteome, the line corresponding to set 3 (nonpathogens) is lower than those of sets 1 and 2 for peptides found rarely in the human proteome, indicating that this group was, as a whole, more similar to the human proteome. However, the length of the error bars indicates that there was considerable variability among the proteomes in each set, particularly for the rarest 5-mers. Because rare peptides are of interest in this study, Figure 2 represents only the region 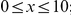 bacterial 5-mers occurring more than ten times in the human proteome are not represented. This is the reason that the sum of the *y*-values does not equal 100% for a given set. The figure suggests that there may be a difference in similarity to human between pathogenic and nonpathogenic bacteria, with pathogenic ones being less similar.

To determine whether there was a significant difference in rare 5-mer frequency among sets 1, 2, and 3, a Kruskal-Wallis test was performed. No significant difference was found (*p*-value 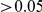 for all three definitions of a rare 5-mer). In addition, the pairwise Wilcoxon rank-sum test was performed between sets 2 and 3, which also revealed no significant differences (*p*-value 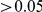 for all three definitions). As explained in the Methods section, these tests were used in lieu of their parametric analogues because the distribution of bacterial similarity appeared non-normal (Figure 1). However, it should be noted that in this case, as well as the majority of other cases in this paper, the corresponding parametric tests resulted in the same statistical decision.

**Figure 3:**
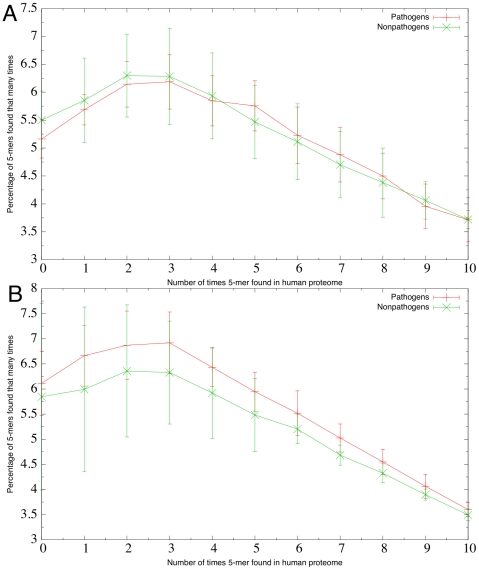
Similarity to the human proteome of surface-accessible proteins from pathogens and nonpathogens. The similarity to the human proteome is shown for proteins (A) from Gram-positive bacteria that are predicted to localize to the cell wall, and (B) from Gram-negative bacteria that are predicted to localize to the outer membrane. Bacterial 5-mers that were found more than ten times in the human proteome are not represented. The length in one direction of the error bar associated with each point represents the standard deviation of the measurements that were averaged to calculate that point.

Since any difference in similarity to the human proteome between pathogenic and nonpathogenic bacteria was visually suggested but not statistically significant, we decided to examine whether there was any difference in similarity among proteins with different subcellular localizations from each set. Specifically, we hypothesized that proteins that are accessible to the immune system (cell wall-localized proteins in Gram-positive bacteria and outer membrane-localized proteins in Gram-negative bacteria) may be more similar to the human proteome in pathogens than in nonpathogens. Figure 3A suggests that, for the “zero-times” and “two or fewer times” definitions of a rare 5-mer, proteins localized to the cell wall in Gram-positive pathogens were more similar to the human proteome than those in Gram-positive nonpathogens, although this difference was not statistically significant (Wilcoxon rank-sum test; *p*-value 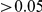 for all three rare 5-mer definitions). The visual suggestion that the pathogenic group was more similar to the human proteome than the nonpathogenic group is notable, however, given that for every other cellular localization, either the nonpathogens were more similar to the human proteome, or the similarity of the two groups was nearly identical (see Figure S1 for scatterplots for all cellular localizations).

Figure 3B shows that the above hypothesis was not supported by the outer membrane proteins in Gram-negative bacteria, for which the nonpathogens appeared more similar to the human proteome than the pathogens. However, this scatterplot is somewhat misleading because there were just four Gram-negative nonpathogens, and one of these–*Rhodobacter sphaeroides*–had few proteins classified as outer membrane relative to the other Gram-negative nonpathogens, but these proteins were extremely similar to the human proteome (just 3.6% of their 5-mers were found zero times in the human proteome). To circumvent this bias, we also tried an alternate visualization method wherein bacteria with few proteins for a specific subcellular localization were not weighted the same as bacteria with many proteins. This method was simple: all the proteins from the Gram-negative nonpathogens were aggregated into a single “pan-proteome”, and likewise with the Gram-negative pathogens. The relative similarity of these two pan-proteomes was then visualized. The resulting scatterplot showed the opposite trend–the pathogens were more similar than the nonpathogens (Figure S2). Note that this scatterplot does not contain error bars, because no averaging was done to obtain the *y*-values–rather, the values were simply calculated on the basis of all the proteins in each pan-proteome.

### Comparison between Acute Pathogens and Chronic Pathogens

**Figure 4:**
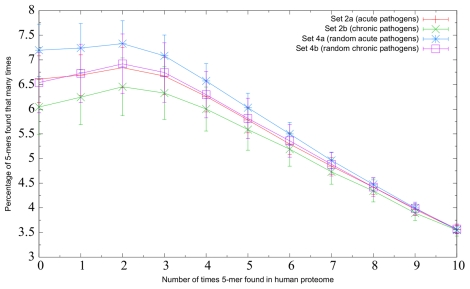
Acute pathogens versus chronic pathogens. Relative similarity to the human proteome of the bacteria in set 2 a (bacteria that cause acute infections), set 2 b (bacteria that cause chronic infections), set 4a (one randomly-generated bacterial proteome corresponding to each proteome in set 2 a ), and set 4b (one randomly-generated bacterial proteome corresponding to each proteome in set 2 b ). Bacterial 5-mers that were found more than ten times in the human proteome are not represented. The length in one direction of the error bar associated with each point represents the standard deviation of the measurements that were averaged to calculate that point.

As described in the Methods section, set 2 was subdivided into set 2 a (acute pathogens) and set 2 b (chronic pathogens) in order to compare their respective similarities to the human proteome. The similarity to the human proteome of these two sets is shown in Figure 4.

Figure 4 suggests that there were significant differences in similarity among the four sets depicted (Kruskal-Wallis test; *p*-value 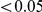 for all three rare 5-mer definitions). In particular, the bacteria causing acute infections contained substantially more rare 5-mers than the bacteria causing chronic infections. This difference was statistically significant for the zero-times definition of a rare 5-mer, and nearly significant for the two or fewer times definition and the five or fewer times definition (Wilcoxon rank-sum test; *p*-value 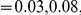 and 0.15, respectively). Furthermore, four of the five pathogens with the smallest proportion of 5-mers found zero times in the human proteome (i.e. more similar) cause chronic infections, and four of the five pathogens with the largest proportion (i.e. less similar) cause acute infections.

We also wondered whether differences in amino acid frequencies in the acute bacteria compared to the chronic bacteria influenced the above results. If so, it would be expected that a randomly-generated proteome with the same amino acid frequencies as a real proteome would have a comparable level of similarity to the human proteome. To investigate this, we constructed ten versions of sets 4a and 4b, which were randomly-generated proteomes with nearly identical amino acid frequencies to sets 2 a and 2 b, respectively. Since all ten versions were highly consistent in terms of similarity, one version of sets 4a and 4b was arbitrarily selected, and the similarity of these sets is shown in Figure 4.

Figure 4 shows that set 4a and set 4b were both substantially less similar to the human proteome than set 2 a and set 2 b, respectively. According to the Wilcoxon rank-sum test, these differences were significant for all three rare 5-mer definitions for the acute pathogens versus the random acute pathogens, but not for the chronic pathogens versus the random chronic pathogens. This inconsistency in statistical significance is likely not due to any actual difference between acute pathogens and chronic pathogens, but rather because there were only six chronic pathogens compared to 14 acute pathogens. However, Figure 4 suggests that amino acid frequencies did play some role in influencing similarity, as the randomly-generated proteomes for the acute pathogens were less similar than the randomly-generated proteomes for the chronic pathogens.

 As described in the Methods section, we used a second randomization method (shuffling the amino acids in each protein) to generate sets 5a and 5b. Like sets 4a and 4b, ten versions of these sets were generated, all of which had consistent similarity profiles. As expected, the similarity profiles of sets 5a and 5b were nearly identical to those of sets 4a and 4b, respectively, so we do not comment further on these sets. The similarity profiles of representative versions of set 5a and set 5b are depicted in Figure S3.

### Examining Other Variables Possibly Related to Similarity to the Human Proteome

In addition to pathogenicity, several other variables may be related to a given bacterium’s similarity to the human proteome. We investigated three such variables: proteome size, G-C content, and (for pathogens) mode of transmission.

**Figure 5:**
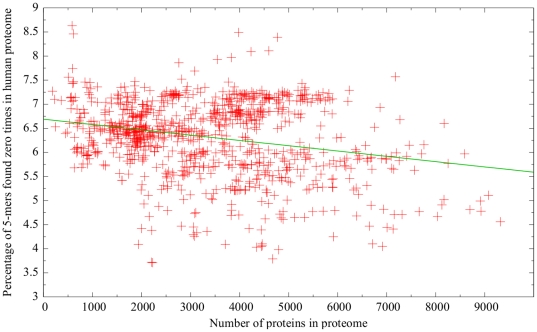
Relationship between proteome size and percentage of 5-mers absent from the human proteome. The best-fit line (in green) was calculated using least squares and had an 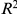 value of 0.062.

Preliminary experiments comparing the similarity of individual bacteria to the human proteome revealed that many of the bacteria that were most dissimilar had severely reduced gene sets. This prompted us to determine whether any general relationship exists between a bacterium’s proteome size and its similarity to the human proteome. Figure 5 shows the relationship between proteome size and similarity to the human proteome for all 975 bacteria in set 1. Overall, these two variables appeared to have little association (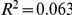). Notable in Figure 5 is the lack of points in the bottom-left corner of the plot–there were no bacteria with small proteomes (fewer than around 1800 proteins) that were highly similar to the human proteome.

**Figure 6:**
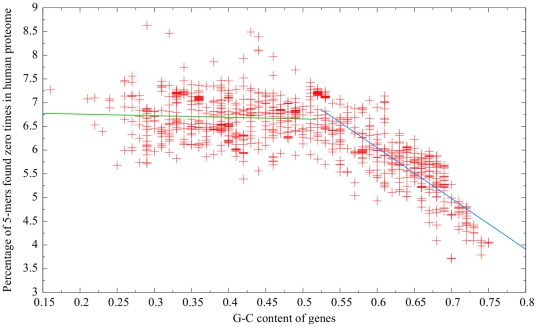
Relationship between G-C content and percentage of 5-mers absent from the human proteome. As the plot exhibits two distinct regions, two best-fit lines were calculated. The green best-fit line was calculated using points with G-C contents less than 0.52 and had an 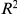 value less than 0.01, whereas the blue best-fit line was calculated using points with G-C contents greater than or equal to 0.52 and had an 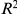 value of 0.74.

Since G-C content influences amino acid composition [17], which in turn seems likely to affect a bacterium’s similarity to the human proteome in terms of rare 5-mer frequency, we investigated the relationship between a bacterium’s G-C content and its similarity to the human proteome. In particular, it seems reasonable to expect that bacteria with G-C contents close to that of the human proteome would also be the most similar at the 5-mer level. Figure 6 shows the relationship between a bacterium’s G-C content and its similarity to the human proteome. If one considers only bacteria with G-C contents less than 0.52, Figure 6 shows that there was essentially no relationship (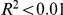) between G-C content and similarity. In contrast, the opposite group of bacteria–those with G-C contents greater than or equal to 0.52 –had a strong association (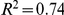). For comparison purposes, using the EMBOSS program *geecee* on the human gene set from the CCDS project website, we determined the overall G-C content of human genes to be approximately 0.52 –interestingly, the approximate point at which the trend appears to change in Figure 6.

Finally, we performed an informal analysis to ascertain whether there were any patterns involving a pathogen’s mode of transmission and its similarity to the human proteome. Unfortunately, we were unable to identify any relationship between these two variables.

## Discussion

In this paper, we made several comparisons that shed light on bacterial similarity to the human proteome. Some of them tested predictions stemming from the hypothesis that there is an inverse relationship between the similarity of a particular peptide to the host, and the host’s ability to make an immune response against it (the “similarity hypothesis”) [1], [2]. The remaining comparisons were designed simply to shed additional light on the matter of proteomic similarity between human and bacteria. In the following subsections, possible interpretations and implications of the data given in the Results section are discussed.

### Characterizing the Range of Bacterial Similarity to the Human Proteome

Table 3 shows the similarity of the ten most-similar and ten least-similar bacteria to the human proteome, along with their G-C contents. It is of interest to conjecture why these bacteria had such extreme similarity or dissimilarity. Before discussing individual bacteria, however, it should be noted that–while the most similar bacteria all had high G-C content and the least similar bacteria all had low G-C content–extremely high G-C content did not guarantee extremely high similarity, and extremely low G-C content did not imply extremely low similarity (see Figure 6). For example, both *Acidimicrobium ferrooxidans* and *Burkholderia cenocepacia* had G-C contents of 0.68, but 4.09% of the former bacterium’s 5-mers were not found in the human proteome compared to 5.88% for the latter.

The dissimilarity of the insect endosymbiont *Blochmannia floridanus* may be due to this organism having an extremely reduced gene set, lacking enzymes for replication initiation, DNA repair, and transcriptional regulation [18], some of which may have homologues in eukaryotes. The same could be said of *Blochmannia pennsylvanicus* [19], as well as *Baumannia cicadellinicola* [20] and *Buchnera aphidicola* [21], two insect endosymbionts with reduced genomes that–while not appearing in the top ten–also had very low similarity to the human proteome. The other bacteria with the lowest similarity to the human proteome were species of the *Bacteroides* genus and the *Eubacterium* genus, both of which are natural inhabitants of the human gut [22], [23].

Curiously, many of the bacteria that were most similar to the human proteome were extremophiles. Two isolates of *Thermus thermophilus*, which grows at 85°C [24], were found in the top ten, as was *Kineococcus radiotolerans*, which can withstand high-level radiation [25]. *Acidimicrobium ferrooxidans* is moderately thermophilic, and is also characterized by its ability to oxidize iron pyrite [26]. Other extremophiles that were not found in the top ten but nonetheless were very similar to the human proteome included *Rubrobacter xylanophilus* (4.3% of 5-mers not found in the human proteome), which is both thermophilic and resistant to gamma radiation [27], and another radiotolerant bacterium, *Deinococcus radiodurans* [28] (4.7%). In contrast to the most similar bacteria, which were either gut flora or were endosymbionts with reduced gene sets, the remainder of the most dissimilar bacteria had no obvious unifying characteristics. The *Anaeromyxobacter* species are characterized as having many genes for surface motility and cellular respiration [29]; *Geodermatophilus obscurus* is found on rock varnish in deserts [30]; *Actinosynnema mirum* is notable for its unique motile spores [31]; finally, *Sanguibacter keddieii* was isolated from the blood of cows [32].

With respect to the similarity hypothesis, it is interesting that the most similar bacteria to the human proteome are extremophiles, none of which are pathogenic. Because they are not pathogenic, other factors must be considered to explain their high level of similarity to the human proteome. The most probable explanation may be that–as with archaea [33]–they share certain cellular processes with eukaryotes. For example, *T. thermophilus*, although classified as a bacterium, has some biosynthetic pathways that have more in common with archaea than bacteria (e.g. [34]), and has also acquired many archaeal genes via horizontal gene transfer [35].

### Comparison between Pathogenic Bacteria and Nonpathogenic Bacteria

The first prediction stemming from the similarity hypothesis tested in this paper was that pathogenic bacteria are more similar to the human proteome than nonpathogenic bacteria. This prediction was based on the rationale that pathogenic bacteria may have selective pressure to evolve to become more similar to their host proteome in order to evade its immune response, whereas nonpathogens would not have this selective pressure. However, the data given in Figure 2 are not consistent with this prediction, as the pathogenic bacteria had a higher frequency of 5-mers that were found rarely in the human proteome–and therefore were less similar to the human proteome using this measure–than the nonpathogenic bacteria.

Figure 2 also shows that the proteomes comprising set 1 (all bacteria) had comparable similarity to the human proteome as those comprising sets 2 or 3. This too was unexpected, as it would seem reasonable to believe that bacteria that can exist in the same environmental conditions as human cells would have a more similar oligopeptide composition than would the set of all bacteria–many of which live in vastly different environments. However, it should be noted that the genomes sequenced to date are heavily overrepresented by bacteria of medical, industrial, or biotechnological importance, and underrepresented by bacteria having a smaller impact on human affairs. As such, the similarity profile of set 1 may not be a faithful representation of all bacteria that exist in nature. The genomes of more diverse groups of bacteria will have to be sequenced before a truly accurate baseline level of bacterial similarity to the human proteome can be established.

Figure 3 and Figure S2 show that, depending on how the data are analyzed, surface-accessible proteins (cell wall-localized proteins in Gram-positive bacteria and outer membrane-localized proteins in Gram-negative bacteria) in pathogens may be more similar to the human proteome than in nonpathogens. In contrast, proteins targeted to other locations either had greater protein-human similarity in nonpathogens, or had comparable similarity in pathogens and nonpathogens. While the possible immunological relevance of this observation needs further study, it is certainly consistent with the similarity hypothesis, which would predict that proteins from pathogens that are accessible to the immune system may experience more evolutionary pressure than other proteins to become similar to the human proteome. Future studies could shed light on this by examining the similarity of pathogen-derived proteins that are actually recognized by the immune system *in vivo*.

Even if there is a biologically relevant relationship between similarity and pathogenicity, our data make it clear that pathogenicity does not always imply high similarity, and that nonpathogenicity does not always imply low similarity. For example, *Legionella pneumophila* is a pathogen, but has lower similarity to the human proteome than the average bacterium. As discussed earlier, *T. thermophilus* is an example of a nonpathogenic species with high similarity to the human proteome. Another example of bacteria that are nonpathogenic but similar are species of the genus *Streptomyces*, which have high G-C content [36]. As such, factors other than pathogenicity must certainly be considered when studying bacteria-human similarity.

### Comparison between Acute Pathogens and Chronic Pathogens

The second prediction was that chronic pathogens are more similar to the human proteome than acute pathogens, with the rationale being that increased similarity could contribute to the enhanced ability of chronic pathogens to evade the immune system. We found that bacteria causing chronic infections were more similar to the human proteome than bacteria causing acute infections (Figure 4), particularly for the zero-times definition of a rare 5-mer. While the actual percentage difference in rare 5-mer frequency may seem rather low–about 0.6 percentage points if you consider 5-mers never occurring in the human proteome–this still translates into a large increase in the absolute number of rarely-occurring 5-mers in acute pathogens compared to chronic pathogens. If a typical bacterial proteome contains 3000 proteins, and each protein contains an average of 300 5-mers, then even 0.6% of the total number of 5-mers is 5400. As immune responses are typically mounted against a small number of immunodominant epitopes [4], this difference may well be large enough to result in a significant selective advantage.

There are at least two plausible explanations for chronic pathogens being more similar to the human proteome than acute pathogens. First, bacteria that are dissimilar to the human proteome may be more likely to cause acute infections, rather than chronic infections, because the immune system is better able to mount a response due to their low similarity. Colloquially, they are forced to hit “hard and fast”, as they are unable to remain in the host for a long time. Another explanation, which is subtly different than the last one, is that acute bacteria are simply unable to cause chronic infections because the body is able to mount an effective immune response, perhaps partially due to their low level of similarity to the human proteome.

Previously, Vider-Shalit et al. [7] found that viruses causing highly acute infections, such as ebola and smallpox, had a higher frequency of peptides likely to be recognized by the immune system than other viruses. Both their result and the data given in this paper suggest that pathogens causing chronic infections may experience greater selective pressure to evade the immune response.

Another interesting feature of Figure 4 and Figure S3 is that the randomly-generated bacterial proteins were much less similar to human than the real bacterial proteomes. This was unsurprising, since the arrangement of amino acids in real proteins is not random, but instead follows patterns related to functional motifs, secondary and tertiary structure, and so on.

### Examining Other Variables Possibly Related to Similarity to the Human Proteome

In addition to factors relating to pathogenicity, the similarity of different bacteria to the human proteome may be affected by a number of other characteristics. Three–proteome size, G-C content, and mode of transmission–were examined in this paper. Although there appeared to be little relationship between similarity and proteome size (Figure 5), it seems noteworthy that no bacteria with small proteomes were among the most similar bacteria to the human proteome. One possible explanation is that bacteria with reduced genomes lack many human homologues that would be present in other bacteria. The relationship between similarity and G-C content was more interesting (Figure 6). Given that the G-C content of human genes was approximately 0.52, it seemed reasonable to expect that the proteomes of bacteria with G-C contents close to 0.52 would exhibit high similarity to the human proteome, and bacteria with G-C contents farther away from 0.52 would be less similar. As such, we hypothesized that as G-C content increased from 0.16 to 0.52, one would expect similarity to the human proteome to increase (since the bacterial G-C content is getting closer to human G-C content); however, the graph indicates no overall change. On the other hand, one would expect similarity to the human proteome to decrease as G-C content increases above 0.52, but the graph shows that, in this region, similarity actually increased in a largely linear fashion. It is difficult to overlook the fact that the boundary between these two sections of the graph occurs at a G-C content level that is approximately the same as the G-C content of human genes. However, this observation is also difficult to explain.

### Future Work

Future work could involve examining more directly the relationship between similarity and immunogenicity. Prior studies have compared the similarity of epitopes and non-epitopes in specific pathogens or even specific proteins [3], [4], and one study [5] compared the similarity of B-cell epitopes from online databases like Bcipep [37], IEDB [38], [39], and AntiJen [40] to surface-exposed oligopeptides in general. However, to our knowledge there has not yet been a study that examines T-cell epitopes from databases like IEDB, AntiJen, and SYFPEITHI [41]. Given that epitopes continue to be discovered at a rapid rate, a reexamination of B-cell data may be warranted; this, combined with an analysis of T-cell epitopes from online databases like those cited above, should give a more thorough picture of the relationship between similarity and immunogenicity.

Another promising avenue for future work would be to test the immunogenicity of peptides as they are mutated to become more or less similar to the human proteome. For instance, the similarity hypothesis would predict that if peptides that are both immunogenic and non-similar were mutated (say, a change of one or two residues) to become similar to the human proteome, then their immunogenicity would be reduced or abrogated. Conversely, peptides that are both non-immunogenic and similar would be predicted to become more immunogenic after being mutated to be less similar to the human proteome. If a large number of peptides in both categories could be tested, and a clear trend consistent with the predictions above was observed, it would strongly support the similarity hypothesis.

There also exists possible future work that is more closely related to that found in this paper. For example, although we compared pathogens with nonpathogens, and acute pathogens with chronic pathogens, other comparisons are possible. For instance, the response against endopathogens and exopathogens is mediated by distinct T helper systems, and the relevance of host-pathogen similarity may be different depending on the type of immune response generated.

Finally, it has recently been discovered that horizontal gene transfer (HGT) can occur between pathogens and humans. Specifically, Anderson and Seifert [42] showed that a fragment of the human long interspersed nuclear element L1 was found in some isolates of *Neisseria gonorrhoeae*–a pathogen that exclusively infects humans. Although little is known about HGT in highly divergent species [42], the fact that it could increase human-pathogen similarity makes it an attractive area of study in the context of the similarity hypothesis. As such, future work could involve identifying and characterizing instances of human-pathogen HGT.

### Conclusions

In this study, we have shown that bacteria-human similarity at the proteomic level varies greatly depending on the bacterial species, with some bacteria having more than twice the frequency of 5-mers not found in the human proteome compared to other bacteria. We also found interesting relationships between bacterial proteome size and similarity to the human proteome, and (in particular) between bacterial G-C content and similarity. Perhaps the most important observations from a translational research perspective are those concerning the relationship between similarity and pathogenicity. In particular, we reported that although chronic pathogens appeared to be more similar to the human proteome than acute pathogens, there was little difference in similarity between pathogens and nonpathogens. In an indirect manner, these observations provide insight into the relationship between immunogenicity and similarity to the human proteome.

Vaccine design studies have traditionally focused on identifying conserved, immunodominant epitopes within pathogens (e.g. [43]-[45]). The identification of such epitopes often involves assays that are time-consuming and expensive, so the ability to predict segments of protein that are promising candidates for inducing an immune response would be very valuable in accelerating the discovery of immunodominant epitopes. The discovery of factors that influence immunogenicity, including peptide-host similarity, could also contribute to increasing the pace of epitope discovery. We hope that the work presented here, as well as related studies, will ultimately provide assistance in this endeavour.

## Supporting Information

Figure S1 **Complete cellular localization results.** Each scatterplot compares the similarity to the human proteome of proteins from pathogens and nonpathogens with a given subcellular localization. Bacterial 5-mers that were found more than ten times in the human proteome are not represented. The length in one direction of the error bar associated with each point represents the standard deviation of the measurements that were averaged to calculate that point.
(PDF)

Figure S2 **Similarity of aggregated sets of outer membrane-localized proteins in pathogens and nonpathogens.** Outer membrane-localized proteins from Gram-negative pathogens were aggregated into a single “pan-proteome”, and likewise for nonpathogens. The similarity to the human proteome of these two pan-proteomes is indicated. Bacterial 5-mers that were found more than ten times in the human proteome are not represented.
(PDF)

Figure S3 **Similarity of shuffled bacterial proteomes.** The relative similarity to the human proteome is shown for the bacteria in set 2 a, set 2 b, set 5a, and set 5b. Bacterial 5-mers that were found more than ten times in the human proteome are not represented. The length in one direction of the error bar associated with each point represents the standard deviation of the measurements that were averaged to calculate that point.
(PDF)

Table S1 **Complete list of bacteria used in this study.** This list corresponds to set 1 as described in the Methods section.
(XLS)
