# Supplementary material for: Comparing the Similarity of Different Groups of Bacteria to the Human Proteome
Source: PLoS One. 2012 Apr 25;7(4):e34007. doi: 10.1371/journal.pone.0034007 (PMC3338800; doi:10.1371/journal.pone.0034007)

Complete cellular localization results

The similarity to the human proteome of the pathogens versus the nonpathogens is shown for proteins having different subcellular localizations.

Proteins with cytoplasmic localization in Gram-negative bacteria

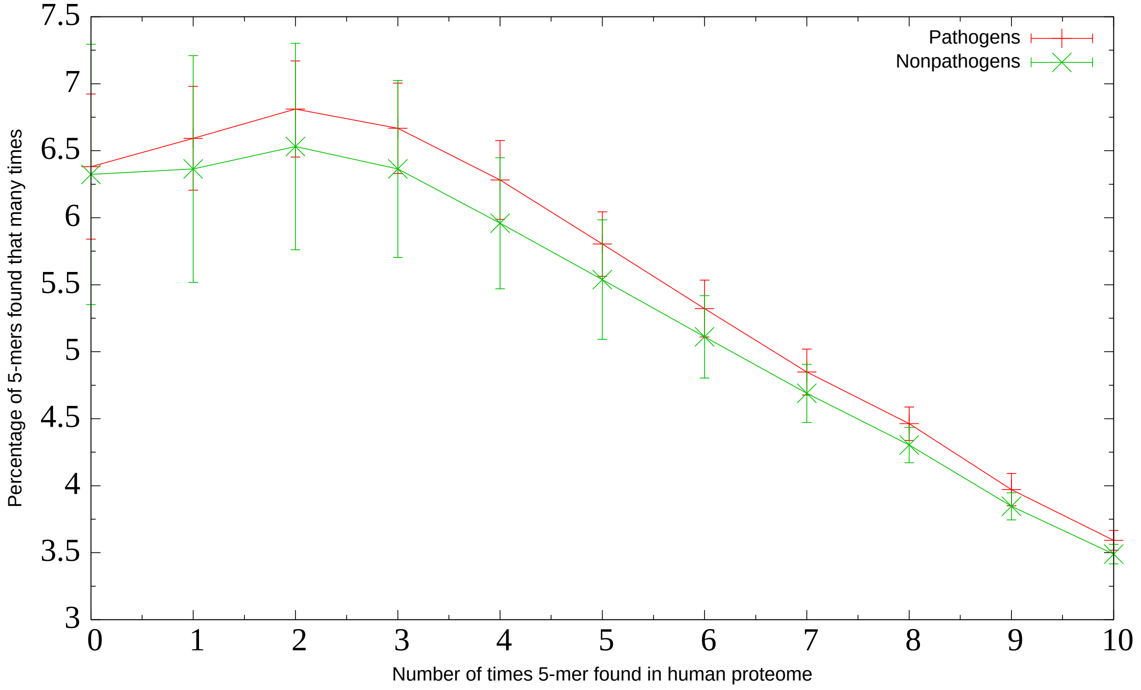

Proteins with cytoplasmic membrane localization in Gram-negative bacteria

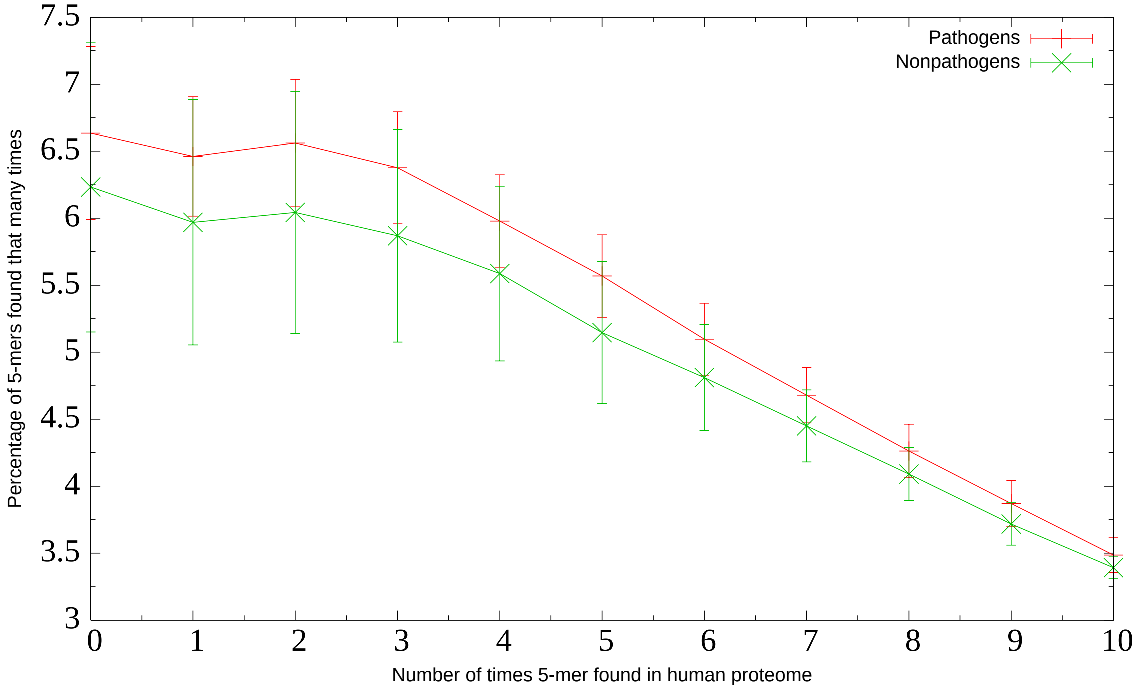

Proteins with extracellular localization in Gram-negative bacteria

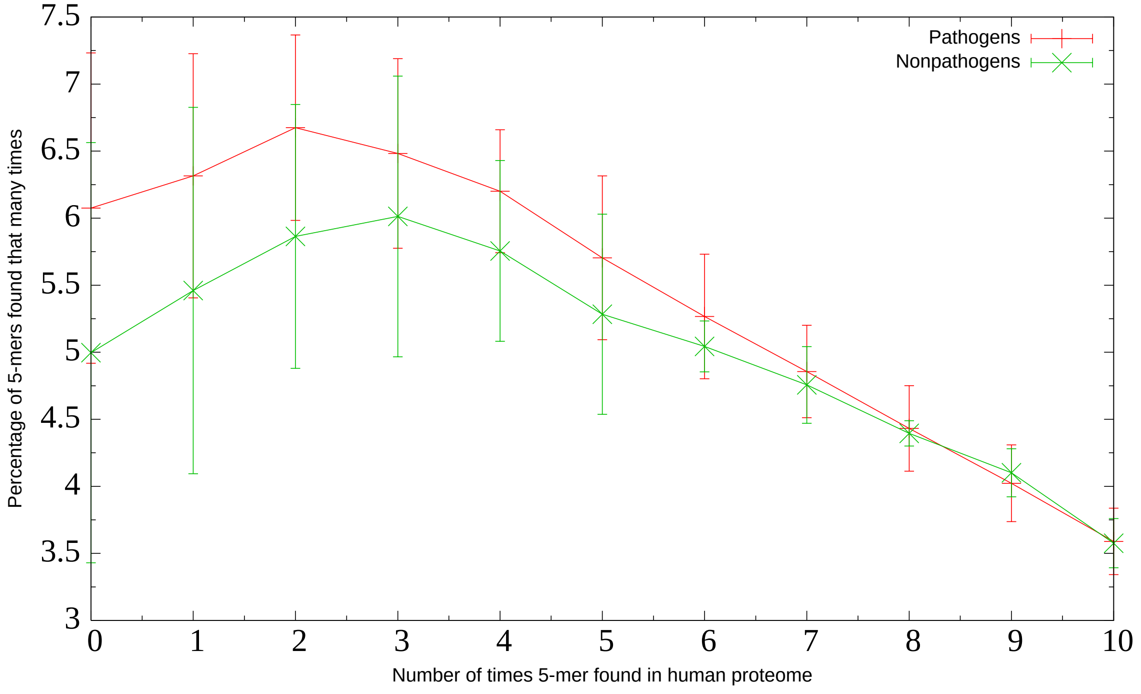

Proteins with outer membrane localization in Gram-negative bacteria

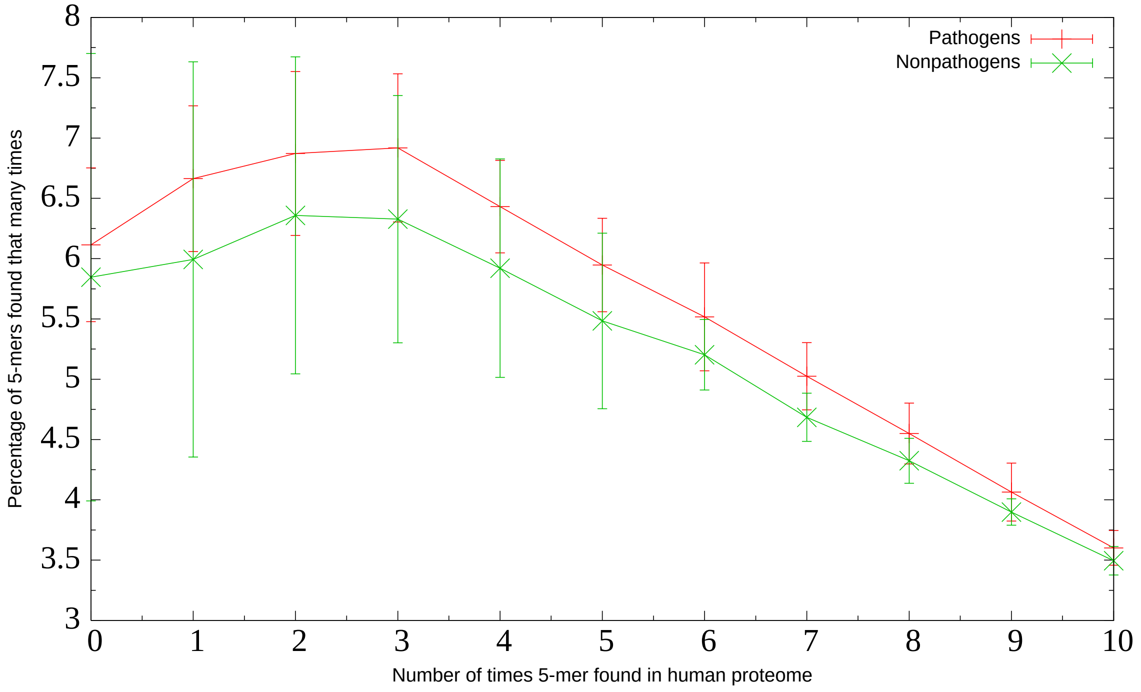

Proteins with periplasmic localization in Gram-negative bacteria

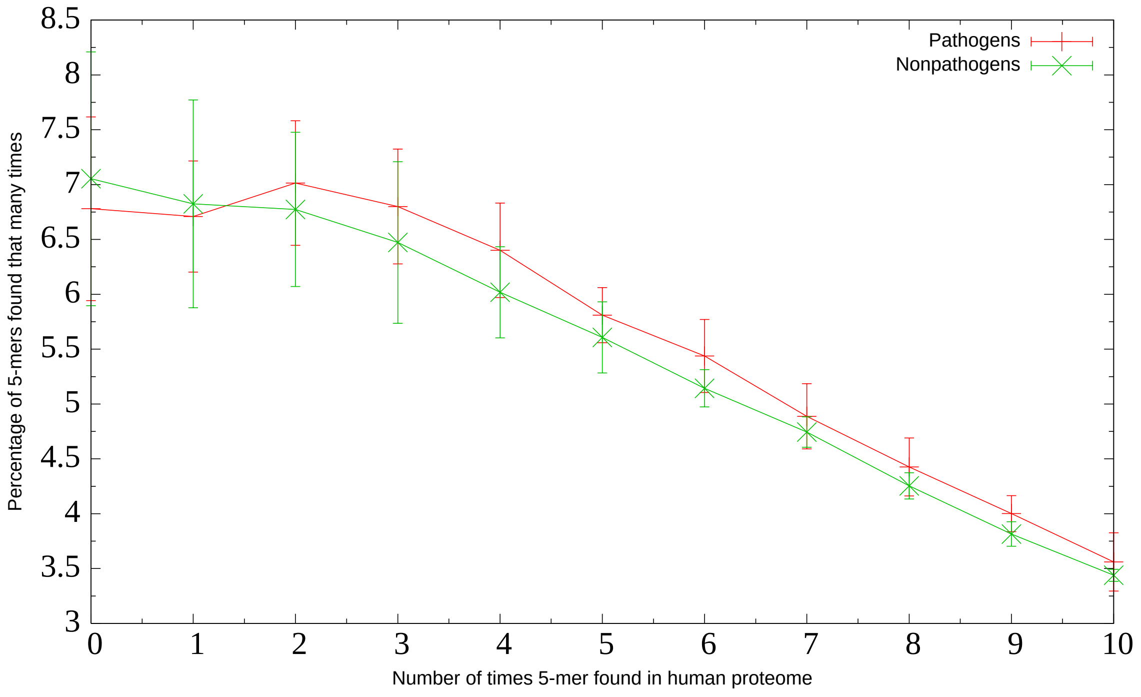

Proteins with cell wall localization in Gram-positive bacteria

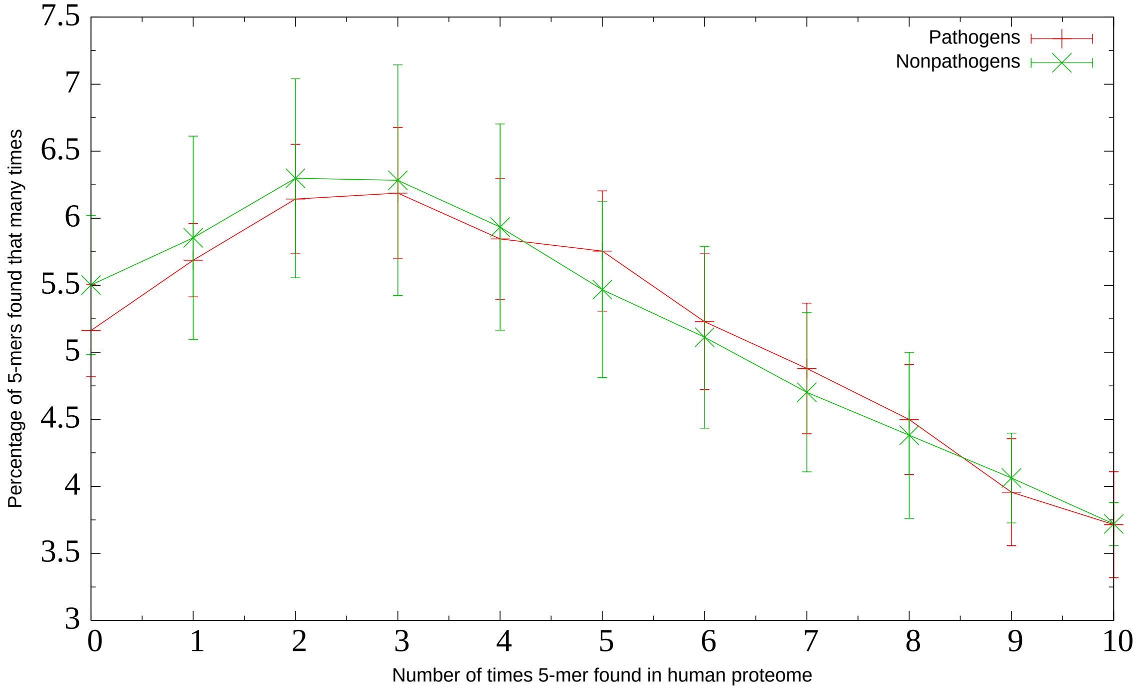

Proteins with cytoplasmic localization in Gram-positive bacteria

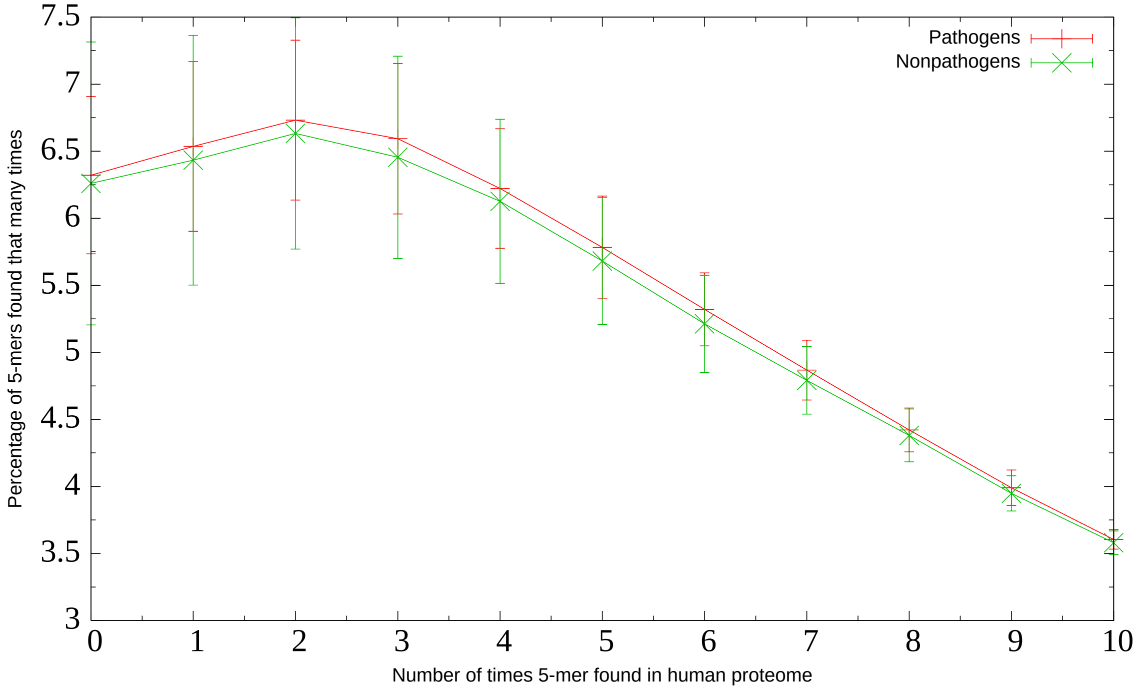

Proteins with cytoplasmic membrane localization in Gram-positive bacteria

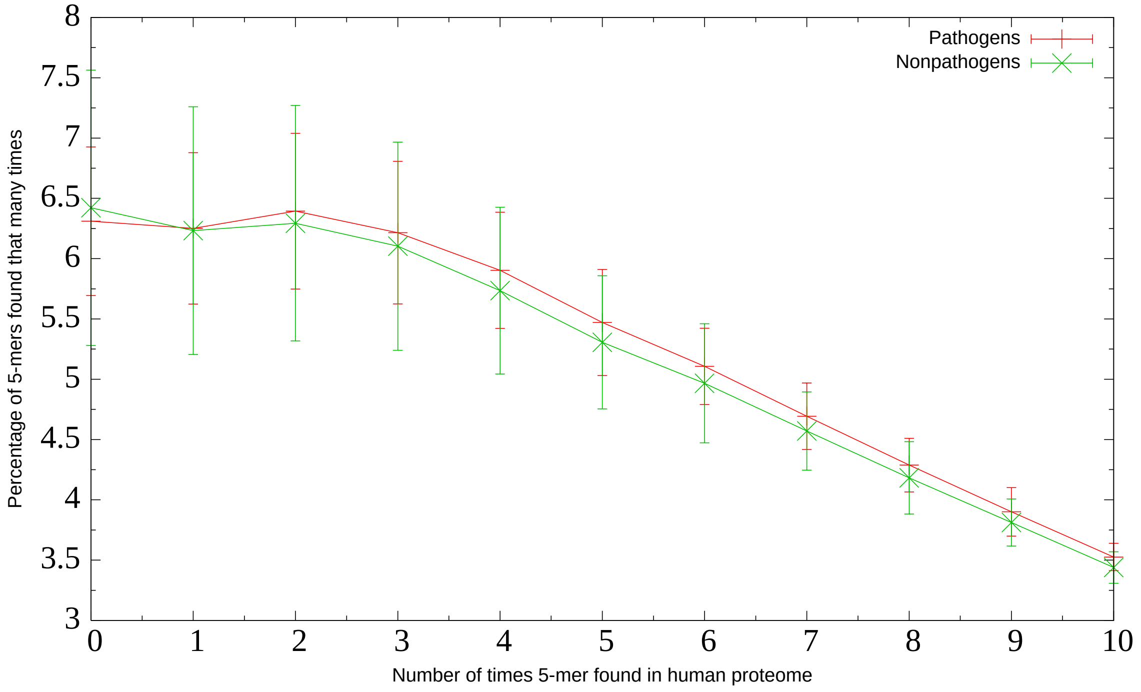

Proteins with extracellular localization in Gram-positive bacteria

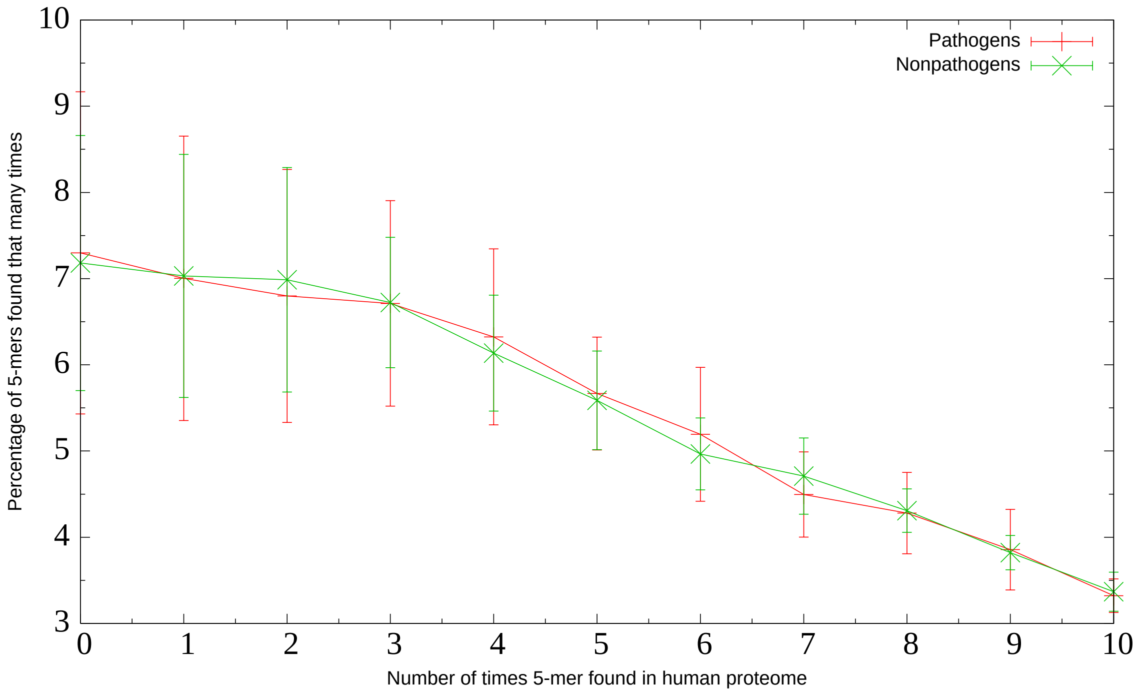

Supplement: Figure S1 — Complete cellular localization results. Each scatterplot compares the similarity to the human proteome of proteins from pathogens and nonpathogens with a given subcellular localization. Bacterial 5-mers that were found more than ten times in the human proteome are not represented. The length in one direction of the error bar associated with each point represents the standard deviation of the measurements that were averaged to calculate that point. (PDF) [file pone.0034007.s001.pdf]
