# Supplementary figures and images for: Comparing the Similarity of Different Groups of Bacteria to the Human Proteome
Source: PLoS One. 2012 Apr 25;7(4):e34007. doi: 10.1371/journal.pone.0034007 (PMC3338800; doi:10.1371/journal.pone.0034007)

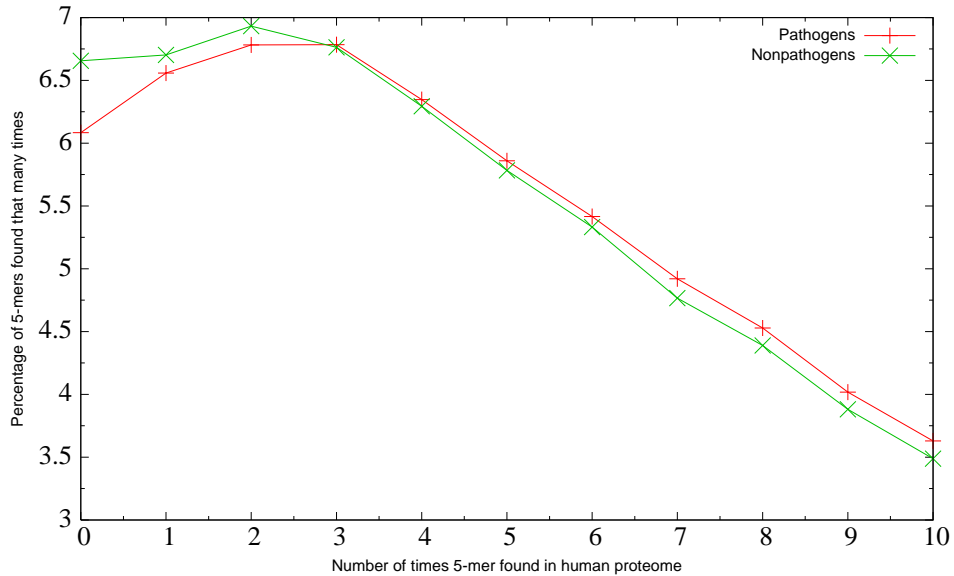

Supplement: Figure S2 — Similarity of aggregated sets of outer membrane-localized proteins in pathogens and nonpathogens. Outer membrane-localized proteins from Gram-negative pathogens were aggregated into a single “pan-proteome”, and likewise for nonpathogens. The similarity to the human proteome of these two pan-proteomes is indicated. Bacterial 5-mers that were found more than ten times in the human proteome are not represented. (PDF) [file pone.0034007.s002.pdf]

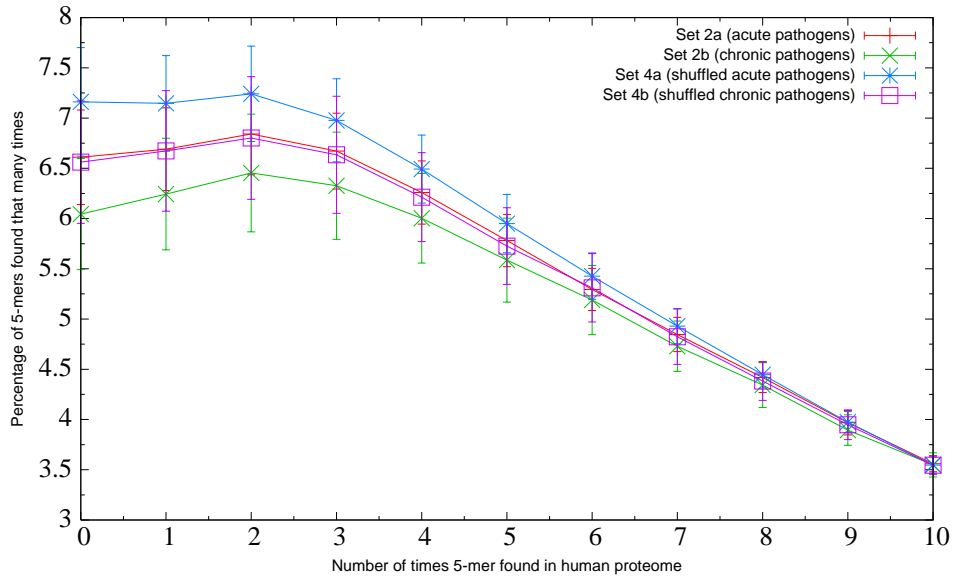

Supplement: Figure S3 — Similarity of shuffled bacterial proteomes. The relative similarity to the human proteome is shown for the bacteria in set 2 a, set 2 b, set 5a, and set 5b. Bacterial 5-mers that were found more than ten times in the human proteome are not represented. The length in one direction of the error bar associated with each point represents the standard deviation of the measurements that were averaged to calculate that point. (PDF) [file pone.0034007.s003.pdf]
